# Incremental Prognostic Value of Conventional Echocardiography in Patients with Acutely Decompensated Heart Failure

**DOI:** 10.5935/abc.20170173

**Published:** 2017-12

**Authors:** Fabio Luis de Jesus Soares, Janine Magalhães Garcia de Oliveira, Gabriel Neimann da Cunha Freire, Lucas Carvalho Andrade, Marcia Maria Noya-Rabelo, Luis Claudio Lemos Correia

**Affiliations:** Hospital São Rafael - Fundação Monte Tabor, Salvador, BA - Brazil

**Keywords:** Heart Failure, Indicators of Morbidity and Mortality, Prognosis, Echocardiography / methods, Hypergravity, Reference Drugs

## Abstract

**Background:**

Acutely decompensated heart failure (ADHF) presents high morbidity and mortality in spite of therapeutic advance. Identifying factors of worst prognosis is important to improve assistance during the hospital phase and follow-up after discharge. The use of echocardiography for diagnosis and therapeutic guidance has been of great utility in clinical practice. However, it is not clear if it could also be useful for risk determination and classification in patients with ADHF and if it is capable of adding prognostic value to a clinical score (OPTIMIZE-HF).

**Objective:**

To identify the echocardiographic variables with independent prognostic value and to test their incremental value to a clinical score.

**Methods:**

Prospective cohort of patients consecutively admitted between January 2013 and January 2015, with diagnosis of acutely decompensated heart failure, followed up to 60 days after discharge. Inclusion criteria were raised plasma level of NT-proBNP (> 450 pg/ml for patients under 50 years of age or NT-proBNP > 900 pg/ml for patients over 50 years of age) and at least one of the signs and symptoms: dyspnea at rest, low cardiac output or signs of right-sided HF. The primary outcome was the composite of death and readmission for decompensated heart failure within 60 days.

**Results:**

Study participants included 110 individuals with average age of 68 ± 16 years, 55% male. The most frequent causes of decompensation (51%) were transgression of the diet and irregular use of medication. Reduced ejection fraction (<40%) was present in 47% of cases, and the NT-proBNP median was 3947 (IIQ = 2370 to 7000). In multivariate analysis, out of the 16 echocardiographic variables studied, only pulmonary artery systolic pressure remained as an independent predictor, but it did not significantly increment the C-statistic of the OPTMIZE-HF score.

**Conclusion:**

The addition of echocardiographic variables to the OPTIMIZE-HF score, with the exception of left ventricular ejection fraction, did not improve its prognostic accuracy concerning cardiovascular events (death or readmission) within 60 days

## Introduction

Acutely decompensated heart failure (ADHF) is a complex and heterogeneous syndrome characterized by the sudden or gradual onset of the signs or symptoms of heart failure, requiring immediate medical attention and treatment.^1^ Mortality reaches 20% within 1 year after the diagnosis, and increases with clinical severity. In those patients with NYHA functional class IV, it can reach 80% within 2 years.^2,3^ The first hospitalization constitutes an important step in the clinical evolution, modifying the quality of life and survival of patients with heart failure.^4^ In spite of the advances in therapeutics, readmission rates due to recurrence of symptoms are high. North American studies in patients over the age of 70 years reveal readmission rates of up to 25%, within 30 days, and 50% within 6 months.^5,6^ Thus, the stratification of patients based on their risk profile for adverse events (such as mortality and HF decompensation) is a crucial task, with a view to improve therapeutic planning and identification of the higher risk subgroup which may benefit from closer monitoring and/or more advanced therapies.^7,8^

Several probabilistic risk models, using clinical variables, have been proposed to predict events in the short and long run.^6,9-12^ Among them is a large registry, the OPTIMIZE-HF^13^ (Organized Program to Initiate Lifesaving Treatment in Hospitalized HF Patients), which provided data on hospital mortality and rehospitalization/death within 60 days after hospitalization using clinical and laboratory variables. In this prognostic model, the only echocardiographic variable tested was left ventricular ejection fraction, computed dichotomously. Other traditional echocardiographic parameters, such as cavity dimensions, left ventricular diastolic function, right ventricular diastolic function, valvular and hemodynamic changes have not been analysed. The association between echocardiographic variables and cardiovascular outcomes, in other studies,^6,14,15^ generates the hypothesis that they may add value to the traditional prognostic models.

As a result, we conducted a study which tests the hypothesis that multiple echocardiographic variables increment the prognostic accuracy of traditional risk prediction by using the OPTIMIZE-HF score.

## Methods

### Population selection

Individuals consecutively hospitalized for ADHF were selected in the cardiac unit of a tertiary hospital, from January 2013 to January 2015. The inclusion criteria for this Registry included individuals with 18 years of age or more and elevated plasma levels of NT-proBNP (> 450 pg/ml in patients < 50 years of age, or > 900 pg/ml in those aged ≥ 50 years), whose hospitalization occurred due to: dyspnea at rest or in the last 15 days; signs of low cardiac output (hypotension - SBP < 90 mmHg; oliguria-diuresis < 0,5 ml/Kg/h; or lowered level of consciousness) or signs of right heart failure (hepatomegaly, lower limb edema or jugular stasis). Pregnant women, patients who did not present adequate acoustic window and those who did not consent to participate in the study were excluded. The protocol is in conformity with the declaration of Helsinki, it was approved by the Research Ethics Committee of the institution and all patients signed a free and clarified consent term.

### Plasma NT-proBNP dosage

The dosage of NT-proBNP was performed on a blood sample collected immediately after the arrival of the patient to the emergency departament, a procedure which aims to ensure the shortest possible time between the beginning of the symptoms and the collection of material. The measurement was performed in serum using the ELFA technique (Enzyme-Linked Fluorescent Assay) and the *bioMerieux VIDAS® NT-proBNP* assay. 

### Transthoracic echocardiography and variables obtained

All the exams have been performed in the first 24 hours after admission in the hospital unit, by only one examiner, blind to clinical and laboratory information. The parameters were obtained in digital format and stored for further analysis, using the GE Vivid 7 machine and the Vivid I system with a M4S sector transducer with frequencies of 1.5 - 3.6MHz. Another trained and qualified observer reviewed the archieved images in 15% of the exams in order to test the interobserver agreement. The patients were studied in left lateral decubitus with sequential analysis of the parasternal, apical, suprasternal and subxiphoid windows. Echocardiographic parameters were assessed in conformity with the recomemndations of the American Society of Echocardiography (ASE).^16,17^ The patients who did not have suboptimal acoustic window, which did not allow satisfactory analysis of the echocardiographic parameters, would not be included in the Registry.

The echocardiographic predictor variables analized were: the left ventricle diastolic diameter, left ventricle systolic diameter, right ventricle diameter, left atrial diameter, left atrial volume (indexed to body surface), tissue Doppler imaging of the tricuspid annulus (S' wave), tricuspid annular plane systolic excursion (TAPSE), left ventricular ejection fraction (Simpson's method), pulsed Doppler analysis of mitral flow (E wave, A wave, E/A relation), lateral and septal mitral annular tissue Doppler (e´septal, e´lateral, S´septal), E/e´relation, systolic pulmonary artery pressure and mitral insufficiency (moderate/severe).

### OPTMIZE-HF predictive model

The OPTIMIZE-HF predictive model, assessed in all patients to admission, involves the collection of clinical and laboratory variables, such as: age, urea, sodium, heart rate, systolic blood pressure and left ventricular systolic dysfunction, in addition to antecedent history of hepatic dysfunction, depression and airway hyperactivity.^13^

### Outcome variable

The primary outcome variable was defined by the composite of death (sudden death or due to HF decompensation) and readmission for ADHF within 60 days.

### Data analysis

#### Statystical analysis

The numerical variables tested were expressed as mean and standard deviation or median and interquartile interval according to normality (Kolmogorov-Smirnov and Shapiro Wilk test), and compared between patients with or without outcome using the unpaired t-test or the Mann-Whitney test. The correlations between the dichotomous variables were performed with the chi-square test. Once outcome-associated variables were identified (p < 0.10), they were inserted into a multivariate logistic regression model, and adjusted according to the OPTIMIZE-HF score. In the final model, variables that proved to be independent predictors (p < 0.05) were added to the OPTIMIZE-HF score. The evaluation of the incremental value of echocardiographic variables was performed by comparing the C-statistic of the model, containing echocardiographic and clinical variables (ECO+OPTIMIZE-HF), with an exclusively clinical model (OPTIMIZE-HF). The areas under the ROC curve were compared using the DeLong test. To evaluate the calibration of the model, the Hosmer-Lemeshow test was performed.

SPSS Statistical Software (Version 21.0, SPSS Inc., Chicago, Illinois, USA) and MedCalc Software (Version 12.3.0.0, Mariakerke, Belgium) were used for data analysis, the latter for comparison between the ROC curves.

#### Sample size calculation

The sample was sized to provide a power of 80% and an alpha of 5%, for the pre-established analysis. To construct a new probabilistic model, in the logistic regression, 1 variable was included for every 5 outcomes. Sample size was calculated to detect a ROC curve with statistical significance, estimating an AUC of 0.75 and an events rate of 25%. A pilot study was carried out with 30 patients and an events rate of 36% of combined outcomes was noted. 110 patients were included, thus allowing for the inclusion of up to 8 echocardiographic variables in a logistic regression model.

## Results

During the period covered by the study, 110 patients diagnosed with ADHF were included. Most patients were elderly people, with an average age of 68 ± 16 years, 55% of them male. Dyspnea was the main symptom in 92% of patients, followed by lower limb edema in 5%. The most common identifiable cause for clinical decompensation was poor drug adherence and/or diet transgression (51%), followed by infection and arrhythmia (21% and 5% respectively). The most common HF etiology was the hypertensive (47%), followed by ischemic heart failure (37%) and Chagas disease (7.2%). The median value of admission NT-proBNP was 3947 (IIQ = 2370 to 7000). The primary outcome occurred in 37 patients (34% of the sample), corresponding to 14 deaths and 23 readmissions within 60 days. The general characteristics are presented in Table 1.

**Table 1:** General Characteristics

	n = 110
Age (years)	68 ± 16
Male	60 (55%)
**Symptom to admission**	
Dyspnea	101 (92%)
Lower limbs edema	6 (5%)
**Decompensation cause**	
Irregular use of medication / Diet transgression	51%
Infection	21%
Arrhythmia	5%
Angina	5%
Digitalis intoxication	3%
Undertemined cause	5%
**HF Etilogy**	
Ischemic	41 (37%)
Hypertensive	52 (47%)
Chagas disease	8 (7.2%)
Valvular	4 (3.6%)
**Comorbidities**	
High blood pressure	82 (75%)
Diabetes Mellitus	49 (45%)
Chronic renal failure	33 (30%)
Previous stroke	17 (16%)
COPD	5 (4,7%)
**Medication in use**	
ACE inhibitors - ARB	77 (70%)
Beta-blocker	53 (48%)
Spironolactone	70 (63%)
Furosemide	40 (36%)
Systolic blood pressure (mmHg)	150 ± 35
Heart rate (bpm)	92 ± 30
Creatine (mg/dl)	1,2 ± 0,6
Urea (mg/dl)	60 ± 30
Sodium (mEq/L)	137 ± 6
LV ejection fraction < 40%	52 (47%)
Admission NT-pro BNP	3947 (IIQ = 237 a 7000)
OPTIMIZE-HF score	35 ± 6
Combined Outcome (death and readmission) within 60 days	37 (34%)
Death within 60 days	14 (13%)
Readmission within 60 days	23 (21%)

HF: heart failure; COPD: chronic obstructive pulmonary disease; ACE inhibitors: angiotensin converting enzyme inhibitors; ARB: angiotensin receptor blocker; LV: left ventricle.

### Echocardiographic characteristics of the studied sample

The echocardiographic analysis has shown that most patients did not present severe left ventricular dilatation, with left ventricular diastolic diameter average of 55.5 ± 11.5 mm. On the other hand, left atrial volume index was significantly raised (47.5 ± 15.6 ml/m^2^).

The analysis of the systolic function has demonstrated that the average left ventricle ejection fraction was 44% ± 17%. In the subgroup of patients with reduced ejection fraction, most of them had severe systolic dysfunction, with a mean LVEF of 29.1% ± 6.5%. It was possible to determine the degree of systolic dysfunction in more than two-thirds of cases, since the other patients presented moderate/severe mitral insufficiency, atrial fibrillation and/or artificial pacemaker stimulation, which could compromise the analysis. From the total individuals evaluated with respect to left ventricular diastolic dysfunction (70 patients), grade I dysfunction (alteration in relaxation) was observed in 28.6% of cases and grades II and III dysfunction (reduced complacency) in 71.4%. However, the estimation of the left ventricular filling pressures was evaluated in all patients using the septal E/e' ratio, and a mean of 23.7±15 was obtained. Estimation of systolic pulmonary artery pressure, through analysis of tricuspid regurgitation, was calculated in all patients, and a mean of 44.4 ± 14.8mmHg was obtained. (Table 2)

**Table 2:** General Chatacteristics

N 110 patients	Averege
LV diastolic diameter (mm)	55.5 ± 11.5
LV systolic diameter (mm)	42.1 ± 14
RV systolic diameter (mm)	30 ± 6.5
LA diameter (mm)	42.6 ± 6.6
Left atrial volume (ml/m²)	47.5 ± 15.6
Tricuspid annular s' wave (cm/s)	12 ± 3.4
TAPSE (mm)	16.8 ± 5
LV ejection fraction (SIMPSOM) (%)	44 ± 17
E wave (m/s)	1.1 ± 0.5
e' septal wave (cm/s)	5 ± 2
Lateral e wave (cm/s)	8 ± 3
Septal E/e'	23.7 ± 15
S' septal wave (cm/s)	5 ± 2
Pulmonary artery systolic pressure (mmHg)	44.4 ± 14.8
Mitral Insufficiency (moderete / severe)	31%
**LV Diastolic Dysfunction**	
Degree I	20 / 70 (28%)
Degree II / III	50 / 70 (71.4%)
IVC diameter (mm)	17.3 ± 5.6
Respiratory variation in IVC (%)	48 ± 30

LV: left ventricle; RV: right ventricle; TAPSE: tricuspid annular plane systolic excursion; IVC: inferior vena cava.

### Echocardiographic predictors

The exploratory analysis of 16 variables, which reflected morphological, functional and hemodynamic changes, was performed, as shown in Table 3. Out of those, only 3 were associated with the primary outcome: left atrial diameter, the indexed volume of the left atrium and the pulmonary artery systolic pressure. The left atrial diameter (44.5 ± 12 mm *versus* 41.8 ± 6 p = 0.05) and the indexed volume of the left atrium (52 ± 17 mm *versus* 45.5 ± 13 mm; p = 0.039) were significantly higher in the events group. With regard to the ejection fraction, there was no statistically significant difference between the groups (44.6 ± 18% versus 43.3 ± 17%; p = 0.72), however when it was examined as a dichotomous, rather than a continuous variable, there was higher prevalence of LVEF < 40% in the outcome group and with statistical significance (61% *versus* 52% p = 0.04). The estimation of left ventricular filling, evaluated through the analysis of the E/e´ relation, did not differ between the two groups (24 ± 13.9 *versus* 23.5 ± 16.7; p = 0.9). However, pulmonary artery systolic pressure was higher in the events group (49.8 ± 14.5 *versus* 42.6 ± 14.7; p = 0.02). The degree of the diastolic dysfunction did not differ significantly between the groups; neither did the presence of moderate/severe mitral insufficiency.

**Table 3:** General characteristics

N 110 patients	Events (37)	Non events (73)	p
LV Diastolic Diameter (mm)	55.6 ± 10	55.7 ± 12	0.94
LV Systolic Diameter (mm)	42 ± 14	42 ± 14	0.84
RV Diameter (mm)	31 ± 6	29 ± 6	0.19
LF Atrial Diameter (mm)	44.5 ± 12	41.8 ± 6	0.05
LF Atrial Volume (ml/m²)	52 ± 17	45.5 ± 13	0.037
RV S' (cm/s)	11.8 ± 3.5	12.1 ± 3.5	0.79
Tricuspid annular plane (TAPSE - mm)	16 ± 5	17 ± 5.1	0.4
LV ejection fraction (SIMPSOM) (%)	44.6 ± 18	43.3 ± 17	0.72
LV ejection fraction < 40%	32 (61%)	38 (52%)	0.04
E wave (m/s)	1.1 ± 0.4	1.1 ± 0.5	0.88
Septal E' (m/s)	0.5 ± 0.21	0.5 ± 0.21	0.68
Lateral E' (m/s)	0.77 ± 0.2	0.8 ± 0.33	0.75
Septal E/e'	24 ± 13.9	17.1 ± 13.3	0.64
Lateral E/e'	15.8 ± 10.2	17.1 ± 13.3	0.64
SPAP (mmHg)	49.8 ± 14.5	46.6 ± 14.7	0.02
**LV Diastolic Dysfunction**			**0.3**
Degree I	11%	18%	
Degree II	27%	24%	
Degree III	19%	22%	
Not possible to grauduate	42%	36%	
Mitral Insufficiency (moderete / severe)	34%	28	0.3

LV: left ventricle; RV: right ventricle; TAPSE: tricuspid annular plane systolic excursion; SPAP: systolic pulmonary artery pressure.

### Clinical and laboratory prognostic predictors

Comparing the non-events group with the events group (death or readmission), no statiscally significant difference was seen in relation to age, sex and systolic blood pressure at admission, as shown in Table 4. In the events group, it was observed that the mean heart rate was significantly higher (99 ± 14 *versus* 89 ± 25; p = 0.04). Lower creatinine level on admission was also noted (1.1 ± 0.5 *versus* 1.4 ±1.3; p = 0.08), but with no statistically significant difference. The OPTIMIZE-HF score was higher in the events group (34.3 ± 7.1 *versus* 29.8 ± 7.2; p = 0.003).

**Table 4:** OPTIMIZE-HF component variables

N 110 patients	Events (37)	Non events (73)	p
Age (years)	72.4 ± 14	68.6 ± 17	0.3
Systolic blood pressure (mmHg)	151 ± 39	146 ± 29	0.6
Heart rate (bpm)	99 ± 14	89 ± 25	0.04
Creatine (mg/dl)	1.4 ± 0.5	1.1 ± 1.3	0.08
Sodium (mEq/L)	138 ± 5	138 ± 6.2	0.9
COPD / Asma	4	18	0.04
CPLD	1	0	0.02
Depression	6	2	0.004
OPTIMIZE-HF	34.3 ± 7.1	29.8 ± 7.2	0.003

COPD: chronic obstructive pulmonary disease; CPLD: chronic parenchymal liver disease.

### Independent and incremental value of echocardiographic variables

In the exploratory analysis, the left atrial volume index and the systolic pulmonary artery pressure (sPAP) were predictors of the primary outcome, and thus selected for multivariate analysis. In the logistic regression, using the OPTIMIZE-HF score and echocardiographic predictor variables, it was observed that the left atrial volume index lost statistical significance, and only the sPAP (p = 0.01) and the OPTIMIZE score (p = 0.002) remained in the final model, as shown in Table 5.

**Table 5:** Univariate analysis: Comparison of clinical-laboratory variables between the events and non-events groups

	Odds Ratio	p
Optimize-HF	1.13 (1.05 - 1.21)	0.002
SPAP	1.05 (1.01 - 1.08)	0.01
Indexed LA volume	1.02 (0.98 - 1.06)	0.4

LA: left atrium; SPAP: systolic pulmonary artery pressure.

**Figure 1:**
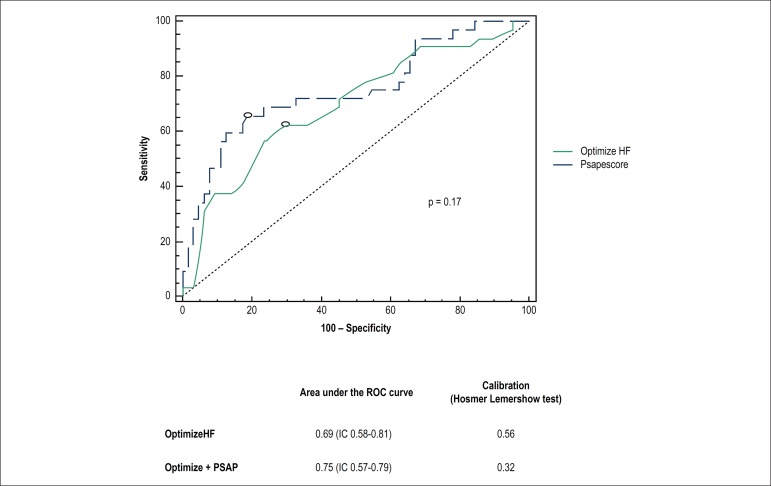
Comparison between ROC curves and C-statistics between the OPTIMIZE-HF conventional and combined (OPTIMIZE-HF+PSAP) probabilistic models, using the DeLong test

The accuracy of the sPAP echocardiographic variable was evaluated using the area under the ROC curve (C-statistic), which resulted in 0.66 (HR 95%; 0.55-0.77), while the area under the curve of the clinical model (OPTIMIZE-HF score) was 0.69 (HR 95%; 0.58-0.81). After sPAP was included in the model, it was observed an increase in the area under the ROC curve to 0.75 (IC 95%; 0.57-0.79). However, this increase was not significant (p = 0.17), which suggests that the echocardiographic variables used did not improve the prediction of events compared to the clinical model, as shown in Figure 1.

## Discussion

The results of this study indicate that routinely measurable echocardiographic parameters, during a standard transthoracic echocardiography, do not seem to improve the risk stratification in patients with ADHF when associated with a clinical score that already uses left ventricular ejection fraction. Only the measurement of pulmonary artery systolic pressure was an independent predictor of death or readmission within 60 days in patients with acutely decompensated HF, but it did not add incremental value to the OPTIMIZE-HF clinical score.

There are several validated prognostic models, each of which combining different variables, which suggests how difficult it is to estimate risks in patients with ADHF. The efforts towards developing and improving such probabilistic models are justified because risk of in-hospital mortality, mortality after discharge and readmission are still elevated in spite of the evolution of specific treatment. The OPTIMIZE-HF score^13^ is one of the tools recommended by the Brazilian Guidelines on Acute Heart Failure,^18^ as well as by other international guidelines^19^ for risk stratification in patients with ADHF. It was developed to evaluate the risk of cardiovascular outcomes in hospital and after discharge (death and readmission). In our sample, the referred score presented regular performance with an area under the curve (AUC) of 0.69 (HR 95%; 0.58 - 0.81; p = 0.002). However, this performance was not significantly improved when echocardiographic variables were added to the score (independent predictor of outcomes), and an AUC of 0.75 (HR 95%; 0.57 - 0.79; p = 0.005) was obtained. This suggests that not all information provided by a negative echocardiogram, or that apparently could indicate a worsen evolution, may improve risk prediction, when evaluated within the context of a clinical score.

The hypothesis according to which echocardiography could have prognostic impact in patients with acutely decompensated heart failure took shape in the late 1990’s, based on a study by Sennim et al.^20^ For the first time, in a population-based study, it was demonstrated that patients with HF who received echocardiographic evaluation had improved survival and were more willing to be treated with angiotensin converting enzyme inhibitors (ACE inhibitors) compared to those patients who were not evaluated by echocardiography. Since then, innumerable echocardiographic variables have been studied and identified as predictors of morbidity and mortality in acute heart failure.^21-28^ Left ventricular ejection fraction is probably the most researched variable and it has been shown to be a predictor of short^29^ and long^30,31^ term mortality in patients with ADHF. In our study, we observed that in those patients who had LVEF < 40%, there were more outcomes when compared to those with LVEF > 40%. However, when we compared the absolute value of LVEF, it did not predict events, which suggests that qualifying the systolic function (LV systolic dysfunction, present or absent) is more important for risk stratification than the numerical value of ejection fraction. Hemodynamic analysis of left ventricular compliance and filling pressures have also been largely studied, based on non-invasive hemodynamic analysis using conventional echocardiography.^32^ The assessment of mitral flow and tissue Doppler allows to infer the therapeutic response in patients with ADHF, since these ratings are directly related to ventricular preload and afterload, which vary considerably in the acute phase of decompensation.^33^ However, available data on the E/e’ relation and its prognostic meaning in the ADHF scenario are few and often conflicting. Some studies assert that this variable is not capable of providing prognostic information on these patients, when admission is evaluated in the emergency unit,^34^ and others suggest that, when it is associated with LVEF, it is possible to identify those patients with higher risk of death and readmission.^30^ In this study, the degree of diastolic dysfunction at admission, in both E/A and E/e´ relations (medial and lateral), were not capable of identifying those patients who had more or less events. Other important component of the echocardiographic analysis of patients with ADHF is the estimation of pulmonary artery systolic pressure. Most of these patients suffer from passive or mixed pulmonary hypertension, that is, a combination of passively elevated pressures and pulmonary vasoreactivity response. These types may improve acutely with blood volume normalization.^35^ Several studies have shown the sPAP as an independent predictor of cardiovascular outcomes.^32,36,37^ In this study, it was observed that sPAP remained as an independent predictor of combined outcomes, even after it was adjusted to the clinical variables that composed the clinical score.

However, the statistical significance in multivariate analysis is not a sufficient requirement to state clinical relevance of the prognostic evaluation. The incremental value in relation to a usual predictive model must also be demonstrated and few studies have incorporated echocardiographic variables to a clinical predictive model and evaluated their performance using the C-statistic increment. Our study has demonstrated that the addition of the 16 (sixteen) echocardiographic variables tested (with the exception of left ventricular ejection fraction categorized as < 40% and > 40% which already composes the OPTIMIZE-HF score) did not improve the prognostic accuracy of the clinical score in predicting cardiovascular events within 60 days. Among the variables tested, the sPAP, with a C-statistic of 0.66 (HR 95%; 0.55 - 0.77) and with p = 0.01 in the logistic regression analysis, was the only one which predicted cardiovascular events within 60 days. However, when it was added to the OPTIMIZE-HF score, the C-statistic increment was not significant. As a result, in spite of its statistical significance in the multivariate analysis, the sPAP was not enough to assert the incremental prognostic value and clinical relevance of the prognostic evaluation in patients with acutely decompensated heart failure. In the review of the literature carried out, we did not find any scientific work that has examined the incremental value of conventional echocardiography to the OPTIMIZE-HF score. A small number of researches has incorporated echocardiographic variables into a clinical predictive model, aiming to evaluate the performance of these variables and their incremental value on the C-statistics of the score tested. Among them, we highlight the research published by Gripp et al.,^38^ which evaluated retrospectively the incremental value of the echocardiography to the ADHERE score, demonstrating that the sPAP added independent prognostic information and allowed a modest increment in the score’s C-statistic, around 0.07, in predicting in-hospital mortality. However, there were no reports that this increase presented statistical significance.

The main limitation of this study is its sample size and the fact that it was carried out in only one center, which means that our data cannot be generalized, nor considered definitive in relation to the lack of prognostic increment in the echocardiographic variables. Another point to highlight is the absence of a second control echocardiography in all the patients, so that the variables could have been compared before and after therapeutic optimization. Echocardiographic variations may occur, such as sPAP decrease in more than 10 mmHg, increase in LVEF from 5 to 10%, reduced degree of mitral insufficiency and/or tricuspid, as well as improved diastolic dysfunction and pericardial stroke. Furthermore, new technologies, such as speckle tracking and three-dimensional echocardiography, were not used, which could have improved the analysis of the biventricular systolic function as well as the cardiac chamber real volumes.

## Conclusion

The addition of echocardiographic variables, except for left ventricular ejection fraction, to the OPTIMIZE-HF score, did not improve its prognostic accuracy in relation to cardiovascular events (death or readmission) within 60 days.
